# Ultra-Processed Foods and Respiratory and Allergic Diseases in Childhood: Epidemiological Evidence and Mechanistic Insights

**DOI:** 10.3390/nu17203269

**Published:** 2025-10-17

**Authors:** Michele Miraglia del Giudice, Giulio Dinardo, Carolina Grella, Alessandra Perrotta, Cristiana Indolfi, Angela Klain

**Affiliations:** Department of Woman, Child and General and Specialized Surgery, University of Campania “Luigi Vanvitelli”, 80138 Naples, Italy; michele.miragliadelgiudice@unicampania.it (M.M.d.G.); dinardogiulio@gmail.com (G.D.); cristianaind@hotmail.com (C.I.); klainangela95@gmail.com (A.K.)

**Keywords:** ultra-processed foods, asthma, children, allergy, food allergy, gut microbiota, inflammation, nutrition, atopic disease

## Abstract

Ultra-processed foods (UPFs) are increasingly consumed worldwide, particularly during childhood, raising growing concerns for health. Although UPFs have been associated with obesity and cardiometabolic disorders, emerging evidence suggests a potential role also in respiratory and allergic diseases. This review critically examines the epidemiological evidence and biological mechanisms linking UPF consumption to respiratory and allergic outcomes in children. To this end, a structured literature search was conducted in the PubMed database, including articles published between 2006 and 2025, selected based on their relevance to the association between UPF consumption and asthma, wheezing, or food allergies in the pediatric population. Four cohort studies on asthma and wheezing, conducted mainly in Brazil and Spain, and two cross-sectional studies—including one global multicenter study—were identified. In addition, four pediatric studies on food allergies from Europe and South America were found, consisting of two cohort studies and two cross-sectional studies. The proposed mechanisms include disruption of the gut barrier, microbiota dysbiosis, chronic inflammation through the AGE–RAGE axis, skewing of immune responses toward a Th2 profile, and indirect effects through obesity and micronutrient deficiencies. Similar pathways may promote allergic sensitization and the development of food allergies. Although current evidence supports the potential role of UPFs in pediatric respiratory and allergic diseases, further longitudinal and interventional studies are needed. Meanwhile, promoting fresh and minimally processed dietary patterns may help protect children’s respiratory and immune health.

## 1. Introduction

Over the past decades, the consumption of ultra-processed foods (UPFs) has risen dramatically across the globe, including in pediatric populations. According to the NOVA classification, UPFs are defined as industrial formulations that contain few or no whole foods and a wide range of synthetic ingredients such as additives, emulsifiers, colorants, flavors, and sweeteners, which are primarily intended to enhance palatability, durability, and market appeal rather than nutritional value [1]. Industrial processing techniques, including the Maillard reaction, hydrogenation, and the formation of advanced glycation end products (AGEs), contribute to the development of compounds with recognized pro-inflammatory and potentially toxic effects [2]. Recent evidence highlights the significant contribution of UPFs to children’s daily caloric intake. In the Canadian CHILD cohort, comprising 2217 children, UPFs accounted for nearly half (45%) of total energy intake by the age of three, and their consumption was positively associated with body mass index and obesity risk at five years of age [3]. An updated meta-analysis further confirmed a dose–response relationship, showing that higher UPF intake significantly increased the risk of overweight and obesity, with odds ratios up to 1.55 [4]. Similar findings were reported in the Spanish longitudinal CORALS study involving 1426 children aged 3–6 years, where greater UPF consumption was linked to higher fat mass, increased waist circumference, elevated fasting glucose, and reduced high-density lipoprotein (HDL) cholesterol [5]. Beyond metabolic outcomes, UPFs appear to exert detrimental effects on child neurodevelopment and immune-related conditions. In the INfancia y Medio Ambiente (INMA) Spanish birth cohort, higher maternal consumption of UPFs during pregnancy was associated with poorer verbal development scores in preschool children [6]. Likewise, a Brazilian study in children and adolescents with food allergy (FA) demonstrated that those with multiple sensitizations had a significantly higher proportion of daily energy intake from UPFs compared with peers with single or no FAs [7]. A comprehensive analysis conducted by the European Academy of Allergy and Clinical Immunology (EAACI) Task Force, which reviewed 55 studies, concluded that UPF consumption is consistently associated with increased risks of asthma, allergic rhinitis, and FA. Proposed mechanisms include disruption of the intestinal barrier, gut microbiota dysbiosis with loss of beneficial taxa, and heightened systemic inflammatory responses [8,9]. Respiratory outcomes may also be influenced: findings from the Seguimiento del Niño para un Desarrollo Óptimo (SENDO) project revealed that higher UPF intake was associated with an increased risk of wheezing in childhood [10]. In contrast, adherence to traditional dietary patterns, such as the Mediterranean diet, has been consistently associated with protective effects against atopic and respiratory diseases. The Mediterranean diet, characterized by a high intake of fruits, vegetables, legumes, fish, and olive oil, has been associated with a reduced risk of asthma and allergic diseases in children, while also promoting favorable metabolic profiles [11,12]. Overall, the growing body of evidence underscores that UPFs are emerging as important determinants of pediatric health (Table 1). Reported effects encompass metabolic, neurodevelopmental, and immune domains, which are relevant for considerations of long-term outcomes. Given the widespread availability and consumption of these products in childhood, there is an urgent need for preventive strategies and the promotion of healthier, traditional dietary patterns early in life. Despite the comprehensive report published by the EAACI Task Force on this topic, important knowledge gaps remain. In particular, the interplay between UPF consumption, genetic background, and other environmental exposures has not been fully addressed. Accumulating epidemiological data provides consistent evidence of an association between UPF consumption and adverse pediatric outcomes. Large birth cohorts, such as the Canadian CHILD study, have shown that UPFs already represent almost half of daily energy intake by the age of three and are prospectively associated with higher BMI and obesity risk at school age. Similar results were reported in the Spanish CORALS longitudinal study, where higher UPF intake was linked to increased adiposity, metabolic alterations, and adverse lipid profiles in preschool children. Importantly, evidence is not limited to metabolic outcomes: cross-sectional and longitudinal pediatric cohorts have consistently reported associations between UPF consumption and asthma, recurrent wheezing, and food allergy. For instance, data from the SENDO project demonstrated that higher UPF intake was significantly associated with wheezing in childhood, while Brazilian and international surveys have linked frequent UPF or fast-food consumption to higher prevalence of asthma, rhinitis, and eczema. Collectively, these findings support a strong epidemiological signal that UPFs may contribute to the burden of respiratory and allergic diseases in childhood, warranting further mechanistic exploration. The aim of the present narrative review is therefore to provide an updated synthesis of the epidemiological evidence linking UPF consumption with respiratory and allergic outcomes in children, while also discussing the potential biological mechanisms involved. By focusing on mechanistic insights and highlighting areas where evidence is still lacking, this review complements previous reports and emphasizes the urgent need for longitudinal and interventional research.

## 2. Ultra-Processed Diets and the Rise of Food Allergies

The escalating prevalence of FA in pediatric populations, particularly within industrialized nations, has prompted intensive scientific inquiry into the environmental drivers underpinning this trend [15]. Parallel to this rise in allergic diseases, there has been a profound shift in dietary patterns, characterized by a significant increase in the consumption of UPFs [7,16,17]. A growing body of evidence now strongly suggests that these dietary shifts are not merely coincidental but are mechanistically linked to the rising incidence of FA [7]. This connection is increasingly understood through the impact of UPF components on gut barrier integrity, immune system programming, and the overall inflammatory state, with dietary AGES emerging as a key mechanistic link [8,9].

Epidemiological studies have highlighted an association between UPF consumption and adverse allergic outcomes. A systematic review conducted by an EAACI task force evaluated evidence from both observational and interventional studies and concluded that dietary exposure to UPFs and their common constituents, including fructose, sugar-sweetened beverages, and multiple food additives, is significantly associated with an increased risk of asthma, allergic rhinitis, and FAs in children [8,10,18,19,20,21,22,23,24]. The review emphasized that these associations were most robust in studies assessing dietary patterns during early childhood, suggesting a critical window of susceptibility. More specifically, evidence from prospective birth cohorts has shown that early introduction of commercial baby foods, many of which are classified as UPFs, is linked to a higher risk of developing challenge-proven FAs during infancy [8,25,26,27,28]. This highlights how dietary exposures in the first years of life may shape immune tolerance or, conversely, promote allergic sensitization. Supporting this notion, a cross-sectional study involving 110 children and adolescents with established FAs demonstrated that a higher contribution of UPFs to total energy intake was significantly associated with both the presence of multiple food allergies and coexisting atopic diseases. These findings point toward a dose-dependent relationship between UPF consumption and the severity and complexity of allergic disease [25]. Consistent with these results, large-scale international surveys provide additional support for the role of dietary patterns in allergy development. The International Study of Asthma and Allergies in Childhood (ISAAC), one of the most comprehensive global investigations into allergic diseases, reported that frequent consumption of fast food (a common subset of UPFs) was positively associated with the prevalence of severe asthma, rhino-conjunctivitis, and eczema in both children and adolescents. These associations were consistent across diverse populations and geographic regions, strengthening the generalizability of the observed link between high-UPF diets and adverse allergic outcomes [8,13,29].

Taken together, the available findings point to a potential link between UPF consumption and the occurrence and severity of allergic diseases in childhood, although the evidence remains limited and largely observational. The consistency of findings across different populations, dietary assessment methods, and disease outcomes underscores the need for further mechanistic research and for dietary guidelines aimed at reducing UPF exposure in children as a preventive strategy against FAs and other allergic conditions.

### Ultra-Processed Foods and Eosinophilic Esophagitis

Emerging hypotheses suggest that UPFs may also be relevant to eosinophilic gastrointestinal disorders, particularly eosinophilic esophagitis (EoE) [30,31,32]. A notable example is the “processed milk hypothesis,” which proposes that industrial processing of cow’s milk markedly increases its allergenic potential [32]. Natural milk contains proteins such as casein and whey that act as natural emulsifiers for fat globules. However, modern techniques, including ultra-heat treatment (UHT) and homogenization, profoundly alter this structure. These processes reduce fat globule size from micrometers to nanometers and denature native proteins, resulting in the formation of lipid–protein nanoparticles that do not occur in raw milk. Such nanoparticles may penetrate the esophageal mucosa more efficiently and act as immune adjuvants, stimulating aberrant immune responses. In particular, they have been hypothesized to promote elevated IgG4 responses against milk proteins including α-lactalbumin (Bos d 4), β-lactoglobulin (Bos d 5), and casein (Bos d 8), pathways that overlap with the Th2- and IgG4-mediated mechanisms central to EoE pathogenesis [32]. It is important to note, however, that no direct epidemiological studies currently link UPF consumption to EoE. This hypothesis highlights the potential role of modern processing in modulating immune recognition of dietary antigens and underscores an important knowledge gap that warrants future investigation [31,32].

## 3. UPF Consumption and Respiratory Diseases

The association between UPF consumption and respiratory diseases has been the subject of investigation both in adults and in children. Brazil has become a leading context for research on UPFs, largely due to the development of the NOVA classification system, its integration into national dietary guidelines, and the availability of large population-based cohorts. The country’s rapid nutritional transition and emphasis on health disparities further support the investigation of UPF-related health outcomes, including respiratory diseases [33,34]. In a population-based cross-sectional study by Serra et al., the association between UPF consumption and asthma prevalence was examined in 1106 Brazilian adults enrolled in the Ribeirão Preto cohort. Dietary intake was assessed using a semi-quantitative food frequency questionnaire (FFQ) previously validated for this population, capturing habitual food consumption over the past year. Foods were classified according to the NOVA system and asthma was identified through self-report of a medical diagnosis. Multivariate logistic regression analyses adjusted for sex, age, income, education, smoking, physical activity, and body mass index (BMI) revealed that individuals in the highest tertile of UPF consumption had 47% greater odds of asthma compared to those in the lowest tertile (adjusted OR = 1.47; 95% CI: 1.01–2.15; p-trend = 0.03). The most frequently consumed UPFs were processed meats, sugar-sweetened beverages, and industrially produced breads [35]. In a large cross-sectional study based on data from the 2012 National Survey of School Health, Pesquisa Nacional de Saúde do Escolar (PeNSE), Melo et al. investigated the association between UPF consumption and respiratory outcomes in 109,104 Brazilian adolescents. Dietary intake of six categories of UPFs, including soft drinks, sweet and salty biscuits, candies, ultra-processed meats, and packaged snacks, was assessed, and participants were classified into quintiles based on an overall UPF consumption score. The prevalence of self-reported asthma and wheezing was 12.4% and 23.2%, respectively. Higher UPF intake was significantly associated with increased odds of both asthma and wheezing in a dose-dependent manner. Adolescents in the highest UPF quintile had 27% higher odds of asthma (OR = 1.27, 95% CI: 1.15–1.41) and 42% higher odds of wheezing (OR = 1.42, 95% CI: 1.35–1.50) compared to those in the lowest quintile. These associations remained robust after adjusting for multiple confounders and were more pronounced among males and those with low fruit and vegetable intake [14].

In recent Spanish research, the authors analysed data from 660 children (mean age 5.1 years) to assess the association between UPF intake and respiratory outcomes. Using a validated food-frequency questionnaire and the NOVA classification, children were divided into tertiles of UPF consumption. Those in the highest tertile (median intake: 305 g/day) had significantly higher odds of wheezing (OR 2.23, 95% CI: 1.11–4.48), recurrent wheezing (OR 2.52, 95% CI: 1.20–5.31), and doctor-diagnosed asthma (OR 2.77, 95% CI: 1.21–6.34) compared to those in the lowest tertile (median intake: 137 g/day). These associations remained significant also after adjusting for multiple confounders, including BMI, physical activity, and parental history of asthma [10].

A recent study by Godbharle et al. investigated the relationship between processed food consumption and respiratory diseases using data from the South African Demographic and Health Survey (SADHS) VII, which included 10,336 adults and adolescents aged 15 and older. The researchers analysed the intake of four categories of processed foods, fried foods, fast food, salty snacks, and processed meats, and their association with eight self-reported non-communicable diseases (NCDs), including asthma and chronic bronchitis. After adjusting for age, sex, and socioeconomic status, the results showed a significant association between salty snack consumption and asthma, with an adjusted odds ratio (AOR) of 1.560 (95% CI: 1.127–2.160). In addition, both salty snacks and processed meats were strongly linked to chronic bronchitis, with AORs of 1.846 (95% CI: 1.133–3.008) and 2.441 (95% CI: 1.506–3.958), respectively. However, no significant associations were found between fried foods or fast food and either asthma or chronic bronchitis after adjusting for confounders [36].

Conflicting results have also been reported. In a prospective analysis using data from the 2004 Pelotas Birth Cohort Study, Machado Azeredo et al. investigated the association between UPF consumption during childhood and asthma outcomes in Brazilian adolescents. The study followed 3493 Brazilian adolescents and assessed their dietary intake at age 6 using a semi-quantitative food-frequency questionnaire. Foods were classified according to the NOVA system, and a UPF score was created by summing the weekly frequency of consumption across 13 predefined UPF categories. Asthma outcomes at age 15 were evaluated through the validated ISAAC questionnaire and included physician-diagnosed asthma, wheezing in the last 12 months, and severe asthma symptoms. Multivariate Poisson regression models were applied to estimate adjusted prevalence ratios (PRs) across quintiles of UPF intake, controlling for potential confounders such as sex, skin colour, family income, maternal education, maternal asthma, household smoking, breastfeeding, and BMI-for-age at 6 years. The results showed no statistically significant association between total UPF consumption at age 6 and any of the asthma outcomes at age 15 in the overall sample. However, in sex-stratified analyses, boys in the highest quintile of UPF consumption had a higher prevalence of physician-diagnosed asthma compared to those in the lowest quintile (PR = 1.35; 95% CI: 1.01–1.81), suggesting a potential sex-specific vulnerability [26].

## 4. Pathophysiological Mechanisms

**Figure 1:**
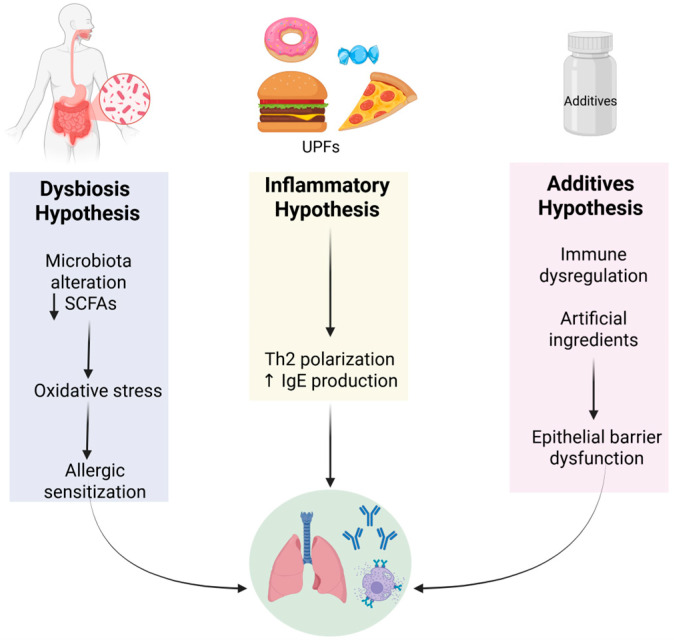
Mechanistic hypotheses linking UPFs to respiratory disease: dysbiosis, inflammation, and immune dysregulation from additives. Created in https://BioRender.com.

UPF consumption has been increasingly connected to the occurrence and characteristics of allergic diseases. The hypothesized mechanisms include alterations in gut microbiota and barrier integrity, chronic systemic inflammation, AGEs production, additive-induced immune responses, obesity-driven endocrine dysfunction, and epigenetic reprogramming [8,30,37,38,39,40,41,42,43,44,45] (Figure 1).

### 4.1. Alterations in the Gut Microbiota and Barrier Function

Diets high in UPFs are devoid of fermentable fibers and bioactive compounds such as polyphenols, which are essential substrates for the growth of beneficial bacterial taxa. This dietary pattern results in a reduction in microbial richness and diversity, favoring dysbiosis and colonization by pro-inflammatory or pathogenic species [38]. One of the consequences of dysbiosis is a reduction in the production of short-chain fatty acids (SCFAs), particularly butyrate, acetate, and propionate. These metabolites are vital for maintaining intestinal epithelial integrity, promoting regulatory T-cell (Treg) function, and suppressing aberrant immune responses. Their deficiency shifts the immune balance toward Th2 and Th17 polarization, increasing susceptibility to allergic sensitization and asthma [39]. Intestinal dysbiosis also weakens the gut and airway epithelial barriers, leading to increased permeability, a phenomenon often described as “leaky gut”. This facilitates translocation of microbial products (e.g., lipopolysaccharides) and dietary antigens into systemic circulation, contributing to low-grade systemic inflammation. Simultaneously, compromised airway epithelial integrity heightens exposure to aeroallergens and pollutants, further exacerbating allergic responses and asthma development. These processes are amplified by the composition of UPFs themselves, which are characteristically rich in refined sugars, saturated fats, sodium, and devoid of anti-inflammatory nutrients like omega-3 fatty acids and antioxidants [40]. This nutrient imbalance triggers chronic activation of innate immune pathways and promotes the systemic release of pro-inflammatory cytokines such as Interleukin-6 (IL-6), Tumor Necrosis Factor alpha (TNF-α), and Interleukin-1 beta (IL-1β), all of which are implicated in airway inflammation, hyperresponsiveness, and tissue remodeling, central features of asthma pathophysiology [39,40].

### 4.2. Role of Advanced Glycation End Products (AGEs)

Another important mechanism is represented by advanced glycation end products (AGEs), a heterogeneous group of compounds formed non-enzymatically when reducing sugars react with proteins or lipids. This process, known as the Maillard reaction, is accelerated by the high-temperature processing methods used to manufacture many UPFs [41]. Preclinical and clinical investigations have demonstrated that these dietary AGEs are not inert; a significant portion is absorbed and contributes to the body’s systemic AGE pool, where they can exert potent biological effects [30,31]. A critical mechanism by which dietary AGEs are proposed to facilitate food allergy is through the disruption of the intestinal epithelial barrier, the gatekeeper of immune tolerance. In vitro studies using human enterocyte models have provided direct evidence that exposure to AGEs significantly compromises gut barrier integrity, reducing transepithelial electrical resistance (TEER) and increasing paracellular flux of molecules [9,42]. This impairment was associated with structural changes, including redistribution and downregulation of tight junction proteins such as occludin and ZO-1. Importantly, this AGE-induced barrier dysfunction has direct immunological consequences, facilitating increased transepithelial passage of major food antigens like β-lactoglobulin (BLG) and ovalbumin (OVA) [9,43]. Such abnormal antigen influx into the sub-epithelial space promotes sensitisation instead of tolerance. Beyond barrier damage, AGEs actively promote a pro-allergic immune environment by acting as “alarmins.” Binding to their receptor RAGE, expressed on epithelial and immune cells, AGEs trigger inflammatory cascades with increased production of Interleukin-25 (IL-25) and Interleukin-33 (IL-33), cytokines that drive Th2 responses [44,45]. This pathway also activates Extracellular signal–regulated kinases 1 and 2 (ERK ½), Nuclear factor kappa-light-chain-enhancer of activated B cells (NF-κB) and increases the production of reactive oxygen species (ROS), fostering oxidative stress and chronic inflammation [37]. Evidence from studies comparing children with and without FA confirms the clinical relevance of these mechanisms, showing higher dietary intake of common AGEs and greater accumulation in skin tissues of allergic patients [9,25]. In addition, other UPF components, such as emulsifiers (carboxymethyl cellulose, polysorbates), have been shown to disrupt intestinal mucus layers, alter the microbiome, and increase permeability, further contributing to risk [8].

### 4.3. Food Additives and Obesity-Driven Epigenetics

Food additives represent another critical factor. Emulsifiers such as polysorbate 80 and carboxymethylcellulose have been shown in animal models to damage epithelial tight junctions and alter microbial communities. Artificial sweeteners, including saccharin and sucralose, disrupt gut microbiota and may impair glucose metabolism and immune regulation. Some additives may also function as haptens, forming neoantigens upon binding with host proteins and initiating hypersensitivity reactions. These immunogenic responses can amplify allergic inflammation and contribute to asthma severity, particularly in predisposed individuals [38]. High UPF intake is also closely linked to obesity, which acts as both a confounder and a mechanistic intermediary in asthma development. Obesity-related asthma represents a distinct phenotype, often characterized by non-eosinophilic airway inflammation, reduced lung compliance, and poor response to corticosteroids. Adipose tissue acts as a metabolically active organ, secreting adipokines such as leptin and pro-inflammatory cytokines, while suppressing anti-inflammatory mediators like adiponectin. This endocrine dysfunction contributes to systemic inflammation and alters immune cell recruitment in the lungs, thereby worsening asthma control and pulmonary function [8]. Moreover, dietary exposures during critical developmental windows, such as in utero or early childhood, may induce long-lasting epigenetic modifications, including Deoxyribonucleic acid (DNA) methylation, histone acetylation, and altered microRNA expression. These mechanisms influence gene regulation involved in immune maturation, lung development, and inflammatory signaling. Prenatal exposure to UPFs through maternal diet may thus prime the fetal immune system toward an allergic trajectory, increasing postnatal asthma risk [46].

### 4.4. Malnutrition and Mechanistic Interplay

Another dimension to consider is the dual burden of malnutrition associated with UPF-rich diets. Despite excessive caloric intake, these diets are often deficient in essential vitamins, minerals, and phytochemicals. This paradox of concurrent overnutrition and micronutrient deficiency compromises innate and adaptive immunity, weakens antioxidant defences, and increases vulnerability to infections and inflammatory diseases, including asthma [8]. Importantly, these mechanisms do not operate in isolation but interact within a dynamic and complex biological network. The synergistic effects of poor dietary quality, gut dysbiosis, immune dysfunction, chronic inflammation, environmental exposures, and genetic susceptibility converge to elevate asthma risk across the lifespan. These effects may be further modulated by sex, age, and socioeconomic status. Importantly, these mechanisms do not operate in isolation but interact within a dynamic and complex biological network. The synergistic effects of poor dietary quality, gut dysbiosis, immune dysfunction, chronic inflammation, environmental exposures, and genetic susceptibility converge to elevate asthma risk across the lifespan. These effects may be further modulated by sex, age, and socioeconomic status.

## 5. Discussion

Emerging evidence highlights UPF consumption as a potential and modifiable correlate of respiratory and allergic diseases in pediatric populations. Beyond the already documented metabolic effects, mechanistic data suggest that UPFs influence the gut microbiota, compromise epithelial barrier integrity, and modulate immune responses, promoting systemic inflammatory states through mechanisms such as the AGE–RAGE axis, the action of food additives, and obesity-related endocrine dysfunction [30,37,38,39,40,41,42,43,44,45,47].

These findings highlight the urgency of integrating dietary quality among the determinants of pediatric health. Pediatric guidelines should consider the degree of industrial food processing as a critical factor, in addition to emphasizing caloric balance and nutritional adequacy [48,49]. In this context, scientific societies and pediatric associations can play a key role in developing clinical recommendations and public health policies aimed at reducing UPF consumption. Simultaneously, the promotion of protective dietary patterns, particularly the Mediterranean diet, appears essential. This regimen, rich in fruits, vegetables, legumes, fish, and extra-virgin olive oil, provides nutrients that modulate oxidative stress, preserve intestinal function, and regulate immune homeostasis, contributing to a balanced gut microbiota [50,51]. Epidemiological evidence confirms that adherence to such dietary patterns reduces the prevalence of asthma, wheezing, and allergic rhinitis in children [12,52,53,54,55]. Children adhering more closely to this dietary pattern show reduced airway inflammation, improved lung function, and fewer allergic manifestations compared with peers consuming Western-type diets. These beneficial outcomes are thought to derive from the synergistic effects of key Mediterranean diet components: antioxidants and polyphenols that counteract oxidative stress, omega-3 fatty acids that dampen pro-inflammatory pathways, and fermentable fibers that enhance gut microbiota diversity and short-chain fatty acid production, thereby modulating systemic and mucosal immunity. Moreover, evidence suggests that regular adherence to this dietary model during critical windows of immune system development may confer long-term resilience against atopic diseases. Together, these findings underscore the Mediterranean diet as a protective model not only for overall health but also as a sustainable, non-pharmacological strategy for the prevention and management of respiratory and allergic diseases in childhood.

From a clinical perspective, incorporating systematic assessments of dietary patterns in children with asthma, FAs, or recurrent wheezing allows for targeted and personalized interventions. Pediatricians and allergists can guide families toward minimally processed dietary patterns, supported by clinical nutritionists integrated into multidisciplinary teams, ensuring the long-term sustainability of interventions [56]. It is also crucial to consider the socioeconomic determinants of UPF consumption, which is more prevalent among disadvantaged families, exposing children to a dual burden of malnutrition and allergic diseases. Public health policies should include incentives to increase access to fresh foods, taxation of ultra-processed products, reformulation of school meals, and regulation of marketing aimed at minors [57]. However, due to the cross-sectional nature of most available studies, causal relationships cannot be established. These associations should therefore be interpreted with caution, considering that multiple dietary, lifestyle, and environmental factors have also changed in parallel with the rise in UPF consumption. Nevertheless, the consistency of findings across diverse populations and dietary assessment methods highlights the need for longitudinal and mechanistic studies, as well as for public health initiatives aimed at promoting healthier dietary patterns in children. Therefore, high-quality prospective and interventional studies are needed to clarify causal relationships and optimize preventive and therapeutic strategies in clinical practice.

## 6. Conclusions

Collectively, current epidemiological evidence, although mainly observational and partly heterogeneous, suggests associations between higher consumption of UPFs and an increased risk of pediatric asthma and allergic diseases. These findings, however, remain insufficient to establish causality, highlighting the need for well-designed longitudinal and intervention studies. Mechanistic data provide biological plausibility, pointing to potential pathways such as microbiota imbalance, impaired epithelial barrier integrity, oxidative stress and inflammation mediated by AGEs, and the effects of food additives and emulsifiers. Nonetheless, such experimental models do not fully reproduce real-life dietary exposures in humans. Evidence from human translational studies, including analyses of biomarkers of exposure and immune activation in pediatric populations, offers further support to these mechanisms, but remains limited. Overall, UPF consumption should be regarded as a potentially modifiable environmental exposure, warranting further investigation through prospective and interventional research to clarify its role in the development of childhood asthma and allergic conditions. Given the emerging but still inconclusive evidence, pediatricians, allergists, and other child health professionals should consider providing balanced counseling to families, encouraging gradual shifts toward minimally processed, Mediterranean-style eating patterns, which have been consistently associated in the literature with favorable respiratory and allergic outcomes, though causality has yet to be confirmed [12,52,53,54,55]. At a broader level, scientific societies and pediatric associations may play a key role in raising awareness and integrating cautious recommendations on limiting UPF consumption within clinical guidance and educational initiatives for healthcare professionals and the public. These organizations are also well positioned to support public health strategies aimed at reducing children’s exposure to UPFs in schools and community settings, and promoting access to affordable, fresh, and nutrient-dense foods [57]. Finally, future research should prioritize high-quality prospective and interventional studies to clarify potential causal pathways and assess the real-world impact of dietary modifications in pediatric populations. A coordinated approach involving clinicians, researchers, educators, and policymakers will be crucial to translate emerging evidence into meaningful preventive actions.

## Figures and Tables

**Table 1:** Summary of health outcomes studied linked to ultra-processed food (UPF) consumption in children. UPFs have negative effects on metabolism, neurodevelopment, risk of allergies, and the respiratory system.

Health Area	Effect of UPFs
Allergies [7,8,13]	More food allergies, asthma, and allergic rhinitis
Respiratory system [10,13,14]	Increased risk of wheezing and breathing problems

## Data Availability

No new data were created or analyzed in this study.
